# Methylene Blue Has Strong Extracellular Virucidal Activity Against a SARS-CoV-2-Related Pangolin Coronavirus with No Intracellular or In Vivo Efficacy

**DOI:** 10.3390/pathogens13110958

**Published:** 2024-11-04

**Authors:** Lai Wei, Yuezhen Ma, Yuhao Ren, Shanshan Lu, Xiumei Xiao, Shengdong Luo, Xiaoping An, Erguang Li, Huahao Fan, Lihua Song

**Affiliations:** 1 College of Life Science and Technology, Beijing University of Chemical Technology, Beijing 100089, China; weilai.chongqing@gmail.com (L.W.); 2023201228@buct.edu.cn (Y.M.); lsswork2014@163.com (S.L.); ananxiaoping@sina.com (X.A.); 2 State Key Laboratory of Pathogen and Biosecurity, Academy of Military Medical Sciences (AMMS), Beijing 100071, China; www2354@126.com; 3 The Department of Laboratory Medicine, Peking University Third Hospital, Beijing 100191, China; xxmlza@163.com; 4 Institute of Infectious Diseases Medicine, The Fifth Medical Center of PLA General Hospital, Beijing 100039, China; lsdwork2010@163.com; 5 State Key Laboratory of Pharmaceutical Biotechnology, Medical School, Nanjing University, Nanjing 210093, China

**Keywords:** methylene blue, nirmatrelvir, SARS-CoV-2, GX_P2V, CAG-hACE2 mouse

## Abstract

Studies have demonstrated that methylene blue exhibits significant antiviral activity against SARS-CoV-2 or related coronaviruses at the cellular level, suggesting its potential as an anti-SARS-CoV-2 drug. Herein, we report that methylene blue does not exhibit noticeable antiviral activity in a lethal model involving SARS-CoV-2-related pangolin coronavirus GX_P2V (short_3UTR) infection in CAG-hACE2 transgenic mice. We employed plaque reduction assays and cell infection experiments to compare the extracellular virucidal activity of the compound and its ability to inhibit viral replication in cells to those of nirmatrelvir. Methylene blue demonstrated strong virucidal activity but did not inhibit viral replication in cells. The control compound nirmatrelvir lacked virucidal activity but exhibited strong abilities to inhibit viral replication. The virucidal activity of methylene blue was further tested in mouse plasma. Incubation in mouse plasma increased the virucidal EC_50_ value of methylene blue, indicating that mouse plasma can affect the stability of the compound, although mouse plasma has some extent of natural virucidal activity. These findings elucidate why methylene blue lacks antiviral efficacy in vivo and provide insights for the development of antiviral drugs.

## 1. Introduction

Methylene blue (MeBlu), a tricyclic phenothiazine compound, exhibits broad-spectrum virucidal activity when exposed to light and is utilized to inactivate viruses in blood products prior to transfusions [1,2,3,4]. Approved by the FDA for treating methemoglobinemia, it has also been explored in various clinical studies [5,6]. Following the outbreak of COVID-19, MeBlu was identified as a potential anti-SARS-CoV-2 agent [7,8,9,10,11,12]. Multiple in vitro studies have indicated that MeBlu has significant antiviral activity against SARS-CoV-2, even in the absence of light, with an effective concentration (EC_50_) ranging from 0.3 μM to 1.7 μM [7,9,10,11]. Pseudovirus experiments have indicated that MeBlu inhibits the interaction between the SARS-CoV-2 spike protein and ACE2 at an inhibitory concentration (IC_50_) of 3.5 μM [8]. These EC_50_ values are well below the cytotoxic concentration (CC_50_ > 100 μM) of MeBlu, indicating a favorable therapeutic effect [9].

In our previous drug repurposing study, we also identified MeBlu as a potential antiviral agent against the SARS-CoV-2-related pangolin coronavirus GX_P2V [13]. Recently, we developed a surrogate SARS-CoV-2 lethal infection model using a GX_P2V-derived attenuated mutant known as GX_P2V (short_3UTR) and the CAG-hACE2 transgenic mouse [14,15]. In this study, we report that MeBlu exhibits no antiviral activity in this surrogate SARS-CoV-2 animal infection model. Further in vitro studies demonstrated that while MeBlu has strong virucidal activity outside cells, it lacks intracellular antiviral activity, and its virucidal activity is reduced by the presence of mouse plasma.

## 2. Results

### 2.1. Methylene Blue Has No Antiviral Activity in a Surrogate SARS-CoV-2 Mouse Model

**Figure 1:**
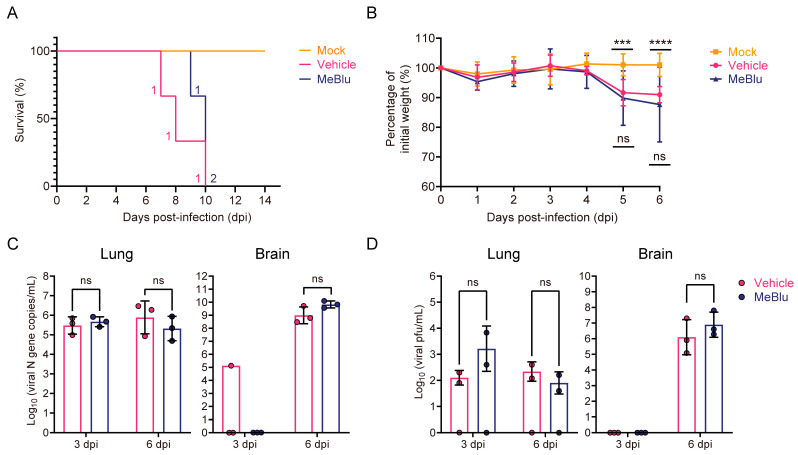
Methylene blue (MeBlu) had no significant prophylactic or therapeutic effect on GX_P2V (short_3UTR) infection in CAG-hACE2 transgenic mice. CAG-hACE2 transgenic mice were orally administered 60 µg/mL MeBlu three days before intranasal challenge with 5 × 10^5^ plaque forming units (pfu) of GX_P2V (short_3UTR). Survival curves (n = 3 per group) (**A**) and percentages of initial body weight change (n = 6 per group) (**B**) of three groups of mice. Significant differences were calculated between the MeBlu group and the vehicle or mock group. The error bars represent the means ± SDs (**B**). (**C**) Viral loads in lung and brain tissues of the sacrificed mice on days 3 (n = 3 per group) and 6 postinfection (n = 3 per group) (3 and 6 dpi). Error bars represent the means of log_10_ (copies/mL) ± SDs. (**D**) Infectious viral titers measured by plaque assay in lung and brain tissues at 3 and 6 dpi. Error bars represent the means of log_10_ (pfu/mL) ± SDs. *p* values were calculated by two-way ANOVA or two-tailed *t* test; *** *p*  <  0.001, **** *p * <  0.0001, ns, not significant (*p* > 0.1).

Previous in vitro findings prompted us to first evaluate the antiviral activity of MeBlu in our surrogate SARS-CoV-2 infection and pathogenesis mouse model [15]. Three days prior to viral infection, MeBlu was added to the drinking water of the mice in the MeBlu group at a final concentration of 60 μg/mL (187.6 μM). This led to the mice exhibiting blue urine, indicating that MeBlu was maintained at a high concentration in vivo. Mice in both the vehicle and mock groups drank normal water. After three days of drinking either water or the MeBlu solution, the mock group underwent a mock infection, while the MeBlu and vehicle groups were infected with GX_P2V (short_3UTR) via intranasal administration. The survival and weight changes among the three groups were monitored daily. The mice in the mock group did not show significant weight loss and survived the entire 14-day observation period without any fatalities. In contrast, the weight of the mice in both the MeBlu and vehicle groups decreased significantly beginning on the fifth day postinfection, and all the mice died by the tenth day (Figure 1A,B). Subsequent analyses using RT–qPCR and plaque assays were conducted to measure viral loads and titers in the lung and brain tissues of the sacrificed mice (Figure 1C,D). The results indicated that on days three and six postinfection, the viral N gene load in the lungs of both the MeBlu and vehicle groups ranged between 5.3 and 5.9 log_10_ (copies/mL). On day six postinfection, viral loads in the brains of the MeBlu and vehicle groups were measured at 9.8 and 9.0 log_10_ (copies/mL), respectively. The plaque assay results were consistent with the qPCR findings, showing no significant difference in viral titers in the brain or lung tissues between the MeBlu and vehicle groups. In conclusion, there was no significant difference in clinical presentation or viral infection outcomes between the MeBlu and vehicle groups, indicating that MeBlu does not exhibit significant antiviral prophylactic or therapeutic activity in this mouse model.

### 2.2. Methylene Blue Has Strong Extracellular Virucidal Activity in the Absence of Light

**Figure 2:**
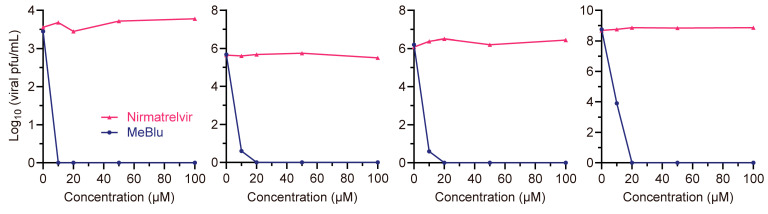
Significant and irreversible extracellular virucidal activity of methylene blue (MeBlu) in the dark. Groups of five concentrations (0, 10, 20, 50, and 100 μM) of nirmatrelvir and MeBlu were incubated with different titers of GX_P2V (short_3UTR) (10^3^, 10^5^, 10^6^, and 10^8^ pfu/mL) for 1 h at 37 °C in the dark, followed by titration using a plaque assay. The results were obtained from four independent experiments.

To understand the antiviral mechanism of MeBlu in vitro, we first used plaque assays to analyze whether MeBlu has extracellular virucidal activity. Nirmatrelvir (an inhibitor targeting the 3C-like protease of SARS-CoV-2) [16] was used as a negative control. Different concentrations of two compounds were incubated with a set number of GX_P2V (short_3UTR) virus particles at 37 °C in the dark for 1 h, after which the infectious virus particles were quantified via a plaque assay after serial dilution (Figure 2). No infectious titers were detected after treatment with 10 μM MeBlu across titers ranging from 3.5 to 6.2 log_10_ (pfu/mL). The titer of GX_P2V (short_3UTR) at 8.7 log_10_ (pfu/mL) decreased to 3.9 log_10_ (pfu/mL) after treatment with 10 μM MeBlu, and no infectious titers were detected after treatment with 20 μM MeBlu. In contrast, the control compound nirmatrelvir did not show significant virucidal activity even at 100 μM (Figure 2). This suggests that MeBlu possesses significant virucidal activity outside of cells, even in the absence of light. The need to highly dilute the virus suspension treated with MeBlu in the plaque assay also indicates that the virucidal effect of MeBlu on GX_P2V (short_3UTR) is irreversible.

### 2.3. Methylene Blue Has No Apparent Ability to Inhibit Viral Replication

**Figure 3:**
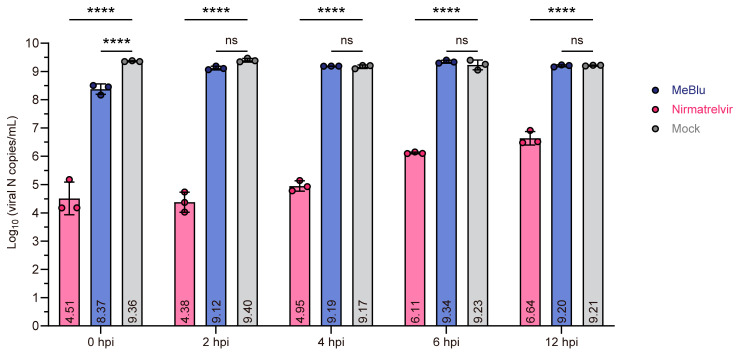
Methylene blue (MeBlu) does not appear to inhibit viral replication. Vero cells were infected with GX_P2V (short_3UTR) at a multiplicity of infection (MOI) of 0.1. At 0, 2, 4, 6, or 12 hpi, the previous culture medium was replaced with fresh medium either without or with 10 μM nirmatrelvir or MeBlu. At 48 hpi, viral N gene copy numbers in culture medium were quantified by RT–qPCR. *p* values were calculated by two-way ANOVA: **** *p * <  0.0001; ns, not significant (*p* > 0.1). The error bars represent the means of log_10_ (copies/mL) ± SDs, and plotted values within the bar represent the means of log_10_ (copies/mL).

Subsequent analysis investigated whether MeBlu and control compound nirmatrelvir could inhibit the replication of GX_P2V (short_3UTR) within cells (Figure 3). Compounds were added to the culture medium at a final concentration of 10 μM at 0, 2, 4, 6, and 12 h postinfection (hpi), and viral N gene copy numbers in the culture medium were analyzed at 48 hpi. Since the highest viral titers in cells infected with GX_P2V (short_3UTR) occurred at 48 h without significant cytopathic effects [14,17], the N gene copy numbers largely reflect the quantity of viral particles. Compared to the untreated control group, adding MeBlu at 0 hpi reduced viral production by approximately 1.0 log_10_ (copies/mL) (90%), but its addition at 2 hpi, 4 hpi, 6 hpi, and 12 hpi had no significant effect on viral output, indicating that MeBlu does not inhibit viral replication. In contrast, nirmatrelvir significantly inhibited viral replication. At 0 hpi or 2 hpi, nirmatrelvir reduced viral production by approximately 4.9 to 5.0 log_10_ (copies/mL), and at 12 hpi, it reduced viral production by 2.6 log_10_ (copies/mL).

### 2.4. Mouse Plasma Exhibits Partial Virucidal Activity but Can Significantly Increase the EC_50_ of Methylene Blue

**Figure 4:**
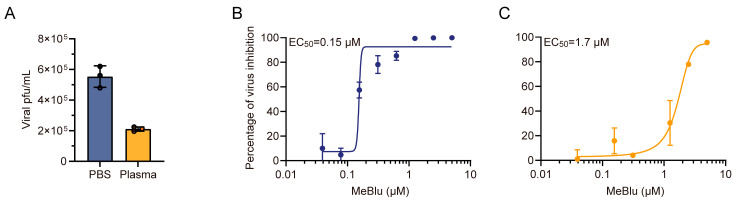
Mouse plasma exhibits partial virucidal activity but significantly inhibits that of methylene blue (MeBlu). (**A**) GX_P2V (short_3UTR) was incubated with plasma or PBS for 1 h in the dark, and infectious viruses were titrated by plaque assay. (**B**) A set of twofold serial dilutions of MeBlu (from 5 µM to ~ 0.039 µM.) was mixed with GX_P2V (short_3UTR). After incubation at 37 °C in the dark for 1 h, the infectious virus was titrated by plaque assay. (**C**) Prior to the above procedure (**B**), fresh and undiluted C57BL/6J Nifdc mouse plasma was incubated with the set of MeBlu for 1 h at 37 °C in the absence of light. The half-maximal antiviral effective concentration (EC_50_) values were calculated via regression analysis using the dose–response curves generated from the experimental data via Prism software (GraphPad Prism 9.3.1.471).

The above results suggested that MeBlu does not inhibit intracellular viral replication but does have strong virucidal activity. However, this does not explain why MeBlu lacks significant antiviral activity in vivo. We further explored the effect of the in vivo environment on the virucidal activity of MeBlu using mouse plasma (Figure 4). Initially, the virucidal activity of the mouse plasma was tested using the plaque assay, and it was found that after incubating the virus in plasma for one hour, the survival rate of the virus was 38.1% (Figure 4A). Subsequently, the effect of mouse plasma on the EC_50_ of MeBlu for inactivating the virus was tested. The results showed that the EC_50_ of MeBlu against GX_P2V (short_3UTR) was 0.15 μM (Figure 4B). However, after incubating MeBlu with animal plasma at 37 °C in the dark for one hour, the EC_50_ value increased to 1.7 μM (Figure 4C). Although both mouse plasma and MeBlu have virucidal activity, their combined incubation significantly increased the EC_50_ of MeBlu (approximately 11 times), suggesting that the virucidal activity of MeBlu is at least partly affected by plasma in vivo, which could partly explain why MeBlu does not exhibit significant antiviral activity in vivo.

## 3. Discussion

Following the outbreak of COVID-19, research on drug repurposing identified several potential antiviral agents at the cellular level, such as MeBlu and ivermectin, all of which lacked reports of in vivo testing. In this study, we utilized a mouse lethal model using the pangolin coronavirus GX_P2V (short_3UTR) to test the antiviral activity of MeBlu. We found that MeBlu did not exhibit antiviral activity against coronavirus in the mouse model. Further analysis revealed that MeBlu does not inhibit viral replication at the cellular level but possesses strong extracellular virucidal activity, which significantly decreases in vivo. Our findings suggest that while MeBlu is a potential extracellular disinfectant against coronaviruses, it is not suitable for use as an antiviral drug.

Previous research reported strong antiviral activity of MeBlu at the cellular level [7,9,10,11], but these studies did not distinguish between extracellular virucidal activity and inhibition of intracellular viral replication. By distinguishing between these two types of activity, our work further deepens the understanding of past reports on the antiviral effects of MeBlu, in the absence of light. Earlier research indicated that MeBlu exhibited nearly 100% inhibition when added four hours prior to infection, whereas the inhibition rate dropped to between 40% and 60% when added two hours post-infection [9,11], findings that align with the observations in our study (Figure 3). Nonetheless, when compared to the control drug nirmatrelvir, this inhibitory effect appears quite modest (Figure 3). Concurrently, a reduction in the infectious viral titer released from cells cultured in media containing MeBlu was observed [10], though a decrease in infectious titer does not necessarily indicate inhibition of viral replication. Earlier studies revealed that MeBlu can inhibit the binding of the spike protein to ACE2 [8], a finding that, combined with our virucidal activity tests, suggests that MeBlu may exert its virucidal activity by binding to the spike protein. Given that MeBlu in the virus suspension is highly diluted in the plaque assay, it is unlikely that it affects the structure of ACE2. This insight helps clarify the mechanisms by which MeBlu may interact with viral components and its potential applications in antiviral strategies. In contrast, in the traditional MeBlu-based photodynamic inactivation of viruses, MeBlu has demonstrated a non-pharmacological virucidal effect within cells [18]. The reactive oxygen species generated by light can physically damage the virus’s nucleic acids and proteins [18,19], which is particularly applicable for superficial treatments of the skin or oral cavity [19].

A reliable drug evaluation system is key in pharmaceutical research. This study used both in vivo and in vitro models based on the pangolin coronavirus GX_P2V (short_3UTR). While SARS-CoV-2 infection in Vero cells results in apparent cytopathic effects [4], GX_P2V (short_3UTR) infection does not cause noticeable cell damage but can release high titers of virus in the culture medium [14]. This characteristic of GX_P2V (short_3UTR) makes it possible to analyze whether MeBlu inhibits viral replication, as viral quantity in the culture medium can be analyzed without the need to consider the viral titer. Additionally, infection of CAG-hACE2 mice with GX_P2V (short_3UTR) leads to 100% mortality [15], allowing for the evaluation of the effectiveness of a drug based on its impact on mouse mortality. In summary, we found that MeBlu has significant extracellular virucidal activity, does not inhibit viral replication intracellularly, and lacks significant antiviral activity in vivo. Our drug evaluation model and methods can serve as a reference for assessing other anti-coronavirus drugs.

## 4. Materials and Methods

### 4.1. Cells, Viruses, and Compounds

Vero (ATCC CCL-81) and BGMK (CVCL_4125) cell lines [14] were propagated in minimum essential medium (MEM) (HyClone, Logan, UT, USA) supplemented with 10% fetal bovine serum (FBS) (PAN Seratech, Eppelheim, Germany) and 1% penicillin/streptavidin (pen/strep) (Gibco, Grand Island, NY, USA). GX_P2V (short_3UTR) was isolated from a pangolin sample in Vero cells and cloned through two successive plaque assays [14,15].

Nirmatrelvir and methylene blue (MeBlu) were purchased from TargetMOI (San Diego, CA, USA). Nirmatrelvir was dissolved in dimethyl sulfoxide, MeBlu was dissolved in nuclease-free water (Invitrogen, Waltham, MA, USA), and the final concentrations were 10 mmol/L (mM).

### 4.2. In Vivo Antiviral Activity Test of Methylene Blue

Six- to eight-week-old C57BL/6J CAG-hACE2 mice were purchased from SpePharm Biotechnology (Beijing, China). As described before [15], the mice were intranasally infected with 5 × 10^5^ plaque-forming units (pfu) of infectious GX_P2V (short_3UTR) or 2% FBS MEM as a mock-infected (mock) group in a 20 µL volume after deep anesthesia by pentobarbital. GX_P2V (short_3UTR)-infected mice were divided into two groups: the MeBlu group, in which drinking water was supplemented with 60 µg/mL MeBlu three days prior to infection, and the vehicle group, in which normal water was provided. Initially, the daily weight and number of deaths in the vehicle group (n = 3), MeBlu group (n = 3), and mock group (n = 3) were monitored. Then, the mice in the vehicle group (n = 3), MeBlu group (n = 3), and mock group (n = 3) were euthanized on the third day postinfection (3 dpi), and an additional series of three groups were euthanized at 6 dpi. Changes in the weights of the mice that were euthanized at 6 dpi (n = 3 per group) were also recorded.

Following euthanasia, the right brain and right lung of the mice were submerged in 400 µL of sterile PBS (HyClone, Logan, UT, USA) and homogenized in a cryomiller. After centrifugation at 12,000 rpm for 10 min at 4 °C, the supernatant was aliquoted and stored at −80 °C for subsequent analysis.

### 4.3. Plaque Assay

BGMK cells from a T175 flask were initially seeded in six-well plates at a ratio of 1:4 one day before the assay. Serial dilutions of GX_P2V (short_3UTR) were added to the cells at a final volume of 500 µL. After infection for 2 h at 37 °C, the BGMK cells were rinsed with PBS and covered with 3 mL of 1% methylcellulose overlay (2 × MEM and 2% methylcellulose mixed at a ratio of 1:1). Five days postinfection, the cells were fixed with 4% paraformaldehyde (Solarbio, Beijing, China) and stained with crystal violet solution (Solarbio, Beijing, China). Finally, the plaques were counted.

### 4.4. Real-Time Reverse Transcription PCR (RT–qPCR) Analysis

Viral RNA was extracted from the supernatant of 200 µL of tissue homogenates or cell cultures using a cell/tissue total RNA extraction kit (Nobelab, Beijing, China). Total RNA was reverse transcribed with a HiScript III RT SuperMix for qPCR (+gDNA wiper) kit (Vazyme, Nanjing, Jiangsu, China). QuantiNova PCR kits (Qiagen, Hilden, Germany) for quantifying the N gene copy numbers was performed in 40 cycles (15 s at 95 °C and 1 min at 60 °C) with the following primers: 5′-TCTTCCTGCTGCAGATTTGGAT-3′, reverse primer: 5′-ATTCTGCACAAGAGTAGACTATGTATCGT-3′ and probe (5′-FAM-TGCAGACCACACAAGGCAGATGGGC-TAMRA-3′).

### 4.5. Plaque Reduction Assay

A volume of 10 μL of nirmatrelvir or MeBlu was added to 190 μL of GX_P2V (short_3UTR) dilutions at different titers (10^3^, 10^5^, 10^6^, and 10^8^ pfu/mL) to obtain final concentrations of each compound of 0, 10, 20, 50, and 100 μM. After incubation in the dark for 1 h at 37 °C, the infectious viruses were titrated by plaque assay.

### 4.6. Antiviral Activity Test in Cells

Vero cells (1 × 10^5^ cells per well) were seeded in 24-well plates one day before the assay. The cells were infected with GX_P2V (short_3UTR) at a multiplicity of infection (MOI) of 0.1 in a final volume of 500 µL for 2 h at 37 °C. After incubation for 2 h in the dark, the media were removed, and the cells were washed twice with PBS. Then, fresh MEM supplemented with 2% FBS was added, and the cells were incubated at 37 °C and 5% CO_2_. For the three treatment groups, the original media were replaced with fresh media containing 10 μM nirmatrelvir or MeBlu at 2, 4, 6, or 12 h postinfection (2, 4, 6, or 12 hpi), respectively. For the 0 hpi group, compounds and viruses were mixed first and then rapidly added to the cell monolayer. The media of the untreated groups were replaced with fresh MEM supplemented with 2% FBS at different times postinfection. Total RNA was extracted from the supernatant of viral cultures 48 h postinfection, and the number of viral N gene copies was quantified by RT–qPCR as described above.

### 4.7. Neutralization Assay in Mouse Plasma

A set of twofold dilutions of MeBlu was first used to determine the effective antiviral concentration of MeBlu against GX_P2V (short_3UTR) in the absence of plasma. A volume of 10 μL of twofold diluted MeBlu solution was added to 190 μL of GX_P2V (short_3UTR) (10^7^ pfu/mL) to dilute the final concentration of MeBlu from 5 µM to ~0.039 µM. After incubating at 37 °C in the dark for 1 h, the infectious viruses were titrated by plaque assay.

C57BL/6J Nifdc mice were purchased from SpePharm Biotechnology (Beijing, China). After deep anesthesia, blood was collected from the orbital sinus of the mice. Whole blood was collected in EDTA anticoagulant tubes (Solarbio, Beijing, China) and centrifuged at 3000 rpm for 5 min to separate the plasma. The plasma of different mice was mixed and aliquoted. A volume of 10 μL of twofold-diluted MeBlu was added to 180 μL of plasma to achieve the same final concentration (from 5 µM to ~0.039 µM) of the compound as mentioned above. Considering the actual situation in the blood during viral infection, we reduced the viral concentration of the test. After the mixture was incubated at 37 °C for one hour, 10 μL of diluted virus was added to a final concentration of 10^5^ pfu/mL. Following another one-hour incubation at 37 °C in the dark, infectious virus concentrations were determined by plaque assay. The virucidal activity of the mouse plasma was analyzed by replacing 10 μL of MeBlu with an equal volume of PBS, which was then incubated with 180 μL of plasma in the dark at 37 °C for 1 h, with 190 μL of PBS serving as the control. After incubating with 10 μL GX_P2V (short_3UTR) in a 37 °C incubator in the dark for one hour, the infectious viruses were titrated by plaque assay.

## 5. Conclusions

This research highlights the importance of understanding the efficacy of potential antiviral drugs against SARS-CoV-2 and its related viruses in vivo, despite their promising in vitro antiviral activities. MeBlu, previously identified as a candidate anti-SARS-CoV-2 agent, shows no antiviral activity in CAG-hACE2 transgenic mice infected with a pangolin coronavirus. Our findings revealed that the antiviral properties of MeBlu are limited to its extracellular virucidal activity and are influenced by factors present in mouse plasma. By comparing the effects of MeBlu with the known antiviral compound nirmatrelvir, we provide insights into the high antiviral activity of nirmatrelvir. This study serves as the first in vivo investigation of MeBlu, offering methodologies and perspectives for future evaluations of antiviral drugs.

## Data Availability

The data that support the findings of this study are available from the corresponding author upon reasonable request.
